# Treatment of Obstruction of the Œsophagus

**Published:** 1897-11

**Authors:** 


### FRENCH.

TRANSLATED BY DR. ALEXANDER GLASS,
PHILADELPHIA, PA.

TREATMENT OF OBSTRUCTION OF THE ŒSOPHAGUS.—Dr. Esmieu recommends the following treatment, which is very simple as well as very efficacious in this malady. It consists of an injection under the skin of the chest of the following solution: Pilocarpine nitrate, 15 cgms.; eserine sulphate, 10 cgms.; distilled water, 5 grms.

A horse, thirteen years of age, and a calf of three months, had obstruction of the œsophagus, due to eating large pieces of potato. These two were completely cured by the hypodermic injection of this solution. In relation to this solution in the horse, Dr. Moussu said it was particularly interesting and useful, as the catheterization of the œsophagus in the horse presented difficulties which were not met with in the same operation in the cow.—*Journal de Médecine, etc.*

